# Changes in facial expressions can distinguish Parkinson’s disease via Bayesian inference

**DOI:** 10.3389/fneur.2025.1533942

**Published:** 2025-03-27

**Authors:** Meimei Mouse, Hongjie Gong, Yifeng Liu, Fan Xu, Xianwei Zou, Min Huang, Xi Yang

**Affiliations:** ^1^Department of Clinic Medicine, School of Clinical Medicine, Chengdu Medical College, Chengdu, China; ^2^Department of Evidence-Based Medicine and Social Medicine, School of Public Health, Chengdu Medical College, Chengdu, China; ^3^Department of Neurology, First Affiliated Hospital of Chengdu Medical College, Chengdu, China; ^4^Department of Physiology, School of Basic Medical Sciences, Chengdu Medical College, Chengdu, China; ^5^Department of Applied Psychology, School of Psychology, Chengdu Medical College, Chengdu, China

**Keywords:** Parkinson’s disease, facial expressions, Bayesian network, tree-augmented network, prediction model, Noldus FaceReader

## Abstract

**Objectives:**

We aimed to clarify the influence of facial expressions on providing early recognition and diagnosis of Parkinson’s disease (PD).

**Methods:**

We included 18 people with PD and 18 controls. The participants were asked to perform 12 monosyllabic tests, 8 disyllabic tests, and 6 multisyllabic tests and the whole process were recorded. Then 26 video clips recorded were used to decipher the facial muscle movements and face expression via Noldus FaceReader 7.0 software. 16 suitable variables were selected to construct a Bayesian network model.

**Results:**

The area under the curve of the unsegmented-syllabic, monosyllabic, dissyllabic, and multisyllabic training models was 0.960, 0.958, and 0.962, respectively, with no significant difference between the models. Based on the Bayesian network models, we found that except for valence in the disyllabic model, all positive facial expressions in the four models are negatively associated with the probability of PD. Moreover, negative facial expressions, including sadness, anger, scared, and disgust in the unsegmented-syllabic, monosyllabic, and multisyllabic models, as well as anger in the disyllabic model, are positively correlated to the probability of PD. Sadness, scare and disgust in disyllabic model are negatively associated with the probability of PD.

**Conclusion:**

Except for sad, scared, and disgusted generated by reading disyllables, negative expressions generated by reading other syllables were positively associated with the probability of PD. In addition, scared expressions produced during monosyllabic reading had the greatest effect on the probability of PD, and disgusted expressions produced during multisyllabic reading had the least effect.

## Introduction

As the global population continues to age, Parkinson’s disease (PD) is taking an increasing toll on productivity and health care resources (1). PD is the second most common neurodegenerative disease and is predicted to double in the coming years (2). The primary manifestations of PD are tremor (3), rigidity (4), bradykinesia (5), postural instability (6), slowness of movement, and ‘masked face’. Hypomimia, means loss of facial expression, often referred to as “masked face,” a typical early symptom, is one of the manifestations of bradykinesia (7). It is characterized by blinking abnormality and reduced spontaneous facial expressions (8).

Researchers have shown that people with PD have trouble expressing basic emotions with their face. In the experiments conducted by Botta et al. (9) motor response during emotional processing was assessed by measuring response times (RTs) in a home-based, forced-choice discrimination task. Participants were asked to discriminate between an emotional stimulus and a neutral one. Additionally, ratings of valence and arousal were also performed. Their findings revealed that PD patients recognize fear as a physical stimulus more quickly. In addition, one of the most studied non-motor symptoms in PD patients is the ability to recognize emotional facial expressions (10). Although the available studies have shown that the people with PD have difficulty recognizing and expressing six basic fascial emotions, their specific impact on the disease is unclear. In our prior study, we found that among PD patients, positive facial expressions decreased while negative facial expressions increased (11).

Our research aims to provide a preliminary foundation for clinical diagnosis and treatment, highlighting the needs and rehabilitation of PD patients with facial expression disorders, to develop more effective facial expression rehabilitation treatment plans. Examples include teaching (non-verbal), communication compensation strategies, speech training, physical therapy, and conference attendance (12). It has been found that “masked face” is considered a contributor to interpersonal and psychological difficulties in PD patients, emphasizing the need for better recognition of the health education requirements of this unique population (13). Smile restoration surgery may be a preferred option for PD patients with severe facial expression disorders (12), contingent on whether the facial muscles are affected or only mildly impacted. Additionally, the severity of “masked face” varies among PD patients at different stages, which was why we have included patients with advanced PD in our study.

The Bayesian network is a machine learning algorithm that involves the creation of probabilistic graph models based on Bayes’ theorem (14). It has numerous advantages in the exploitation of decision support systems, including better interpretability, independent conditional concepts, ease of feature selection, and computational efficiency (15). This method can intuitively represent the complex causal relationship between multiple variables (16), which makes it easier to understand the complex structure and mechanism behind the disease. At present, Bayesian network models are increasingly being used in biomedical and health care to support problem solving, including treatment selection (17), prognostic reasoning (18), diagnostic reasoning (19), and discovering functional interactions (20).

In the process of research, we recruited people with PD and healthy controls. They performed pronunciation tests, and we analyzed their facial expressions using FaceReader software. Noldus FaceReader is a micro-expression analysis software based on facial action units (21). The unique micro-expression analysis module of Noldus FaceReader can automatically analyze 20 commonly used facial action units, respectively measure the activity intensity of the left and right sides of the face and provide important information for facial expression analysis. The non-contact observation method can measure the emotions of the subjects more objectively and accurately without the calibration and intervention of the subjects. The final output consisted of seven expressions (Happy, Sad, Angry, Surprised, Scared, Neutral and Disgusted), of which happy and surprised were positive expressions, and sad, angry, scared, and disgusted were negative expressions (22). In our previous study, we found that in facial movement disorders in PD patients, a decrease in positive facial expressions and an increase in negative facial expressions correlated with muscle stiffness during the progression of Parkinson’s disease (23). In this study, we further confirm the relevant conclusions. Based on this, we hypothesized that an increase or decrease in expression parameters could be used to distinguish between PD and non-Parkinson’s individuals. Therefore, we proposed a Bayesian network model based on facial expression parameters and demographic characteristics to predict PD. We hope that this model could provide a preliminary basis for clinical diagnosis and treatment and help bring attention to the needs and rehabilitation of PD patients with facial expression disorders. For Bayesian data processing, we used R software to filter variables and train the Bayesian Network model, and then used Netica software for further reasoning. The main additions of this publication to previous work are as follows:

1. Netica software is used to construct tree-augmented Bayesian networks.
2. The Bayesian network models are used to predict the probability of PD based on facial expressions and demographic characteristics.
3. Information gain is used to determine the variables to be included in the Bayesian network model.

## Materials and methods

### Participants

We recruited people with PD and healthy controls from the First Affiliated Hospital of Chengdu Medical College from January to August 2019. The inclusion criteria were (23) (1) no previous psychiatric problems or cognitive decline; (2) idiopathic PD with no other neurological impairments; and (3) movement fluctuations that responded well to levodopa. The exclusion criteria were (1) neurological diseases other than PD; (2) significant cognitive impairment that might interfere with speech; (3) severe mental or cognitive impairments that might interfere with speech; (4) clinical problems such as aphasia and severe dysarthria that can affect communication; and (5) participation in other rehabilitation programs or clinical trials.

In total, we recruited 18 people with PD and 18 healthy controls. The people with PD had a confirmed diagnosis of idiopathic PD (according to the UK Parkinson’s Disease Society Bank Criteria). Participants with PD stopped taking levodopa on the morning of the testing to minimize its effect. The instructor conducted systematic training to ensure the consistent quality, including introduced the experimental process to the subjects, and supervised the experimental video recording. In addition, a trained and clinically experienced neuroscientist evaluated and guided the entire project process to ensure that it was performed in an orderly manner.

### Phonation test and face recordings

A vocalization test was used to elicit facial expressions from the participants, to obtain relevant facial expression parameters. Prior to the test, a clinician provided detailed instructions to the participants about the entire process of the vocalization test and facial expression recording, ensuring that all participants could smoothly cooperate and complete the task. To ensure the accuracy of the recorded facial expression data, the examiner guided the participants to complete the task in a relaxed state, without imposing any time constraints. If a participant felt tired, we would pause the test until they were able to complete the remaining portion in a relaxed manner.

**Figure 1:**
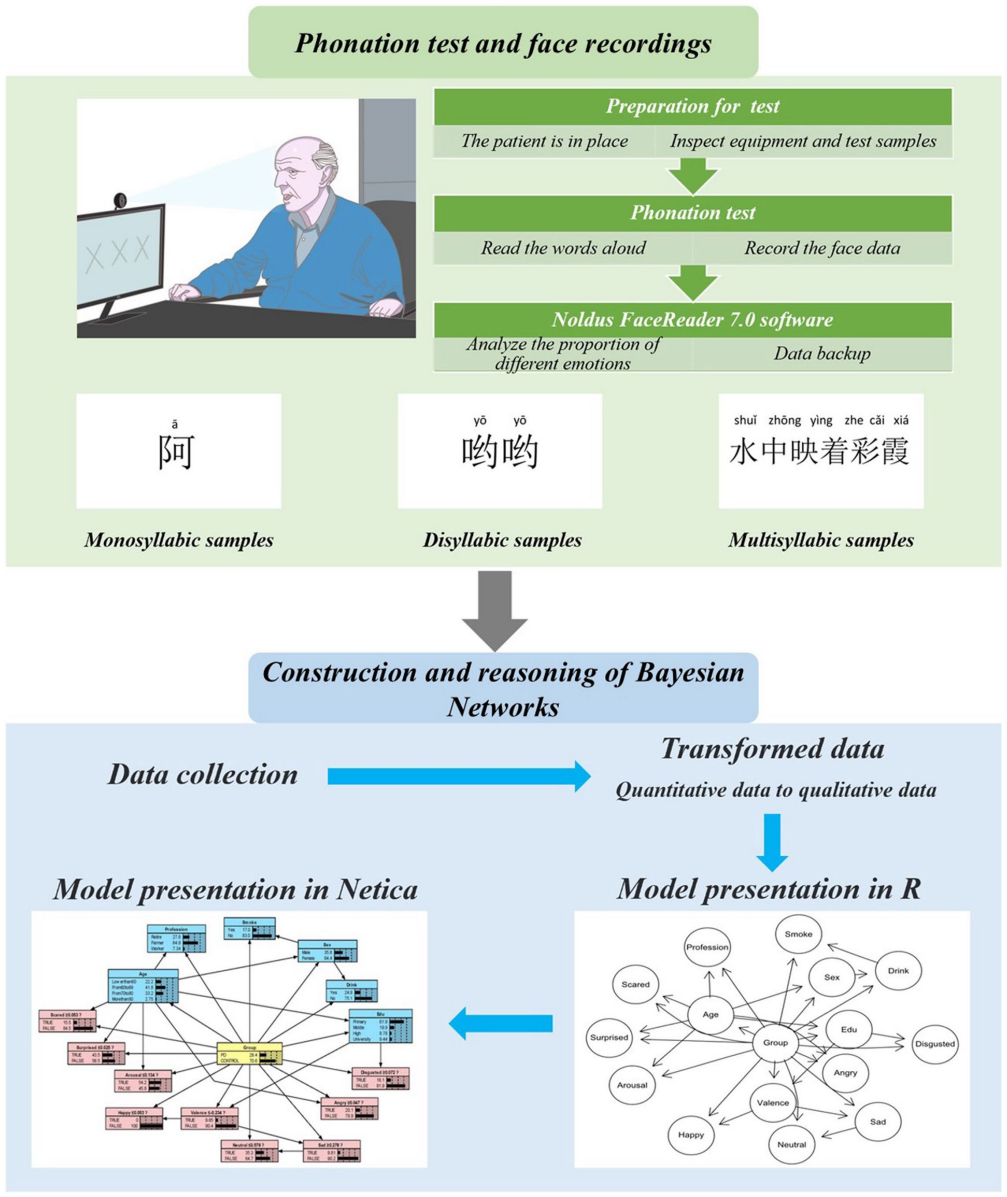
Experimental flowchart.

As shown Figure 1, we designed a PPT consisting of 12 monosyllabic, 8 disyllabic, and 6 polysyllabic words based on the number of local native language. Participants read these syllables and triggered facial muscle movements with pronunciation movements as the trigger point, thus recording their facial expressions. We chose a room with uniform lighting to ensure even illumination of the facial area. Each participant was placed in front of a laptop displaying a slideshow containing the 26 test samples. Simultaneously, the inset front camera of the laptop recorded the entire test process. Participants were instructed to read the content on the slideshow in a relaxed state, maintaining normal tone and volume.

Noldus FaceReader 7.0 software was used to identify the movement of relevant muscle groups during the phonation test. More specifically, it captures the movement of points distributed on the eyebrows, lips and nose, then to infer the changes of face expression (23). While twenty-six recorded video clips were analyzed and coded the facial expressions for the following emotions: neutral, happiness, sadness, anger, surprise, fear, disgust, valence, and excitement accordingly.

### Data collection

We collected a total of 67,224 facial expression parameters. Additionally, we gathered detailed demographic data from all participants, including age, gender, occupation, education level, drinking habits, smoking habits, and other relevant information, which was archived in Excel format.

### Bayesian network

We used 16 random variables (sex, age, drinking, smoking, profession, education level, neutral, happy, arousal, surprised, valence, sad, disgusted, angry, fear and group) to construct the Bayesian network models, which contain a directed acyclic graph (DAG) including the nodes and directed edges. Each node of the DAG corresponds to a variable, and arrows indicate dependence between variables.

### Statistics

The demographic data (gender, occupation, and alcohol consumption) was expressed as frequency (percentage), and was tested using the χ2 test. For continuous variables, first evaluate whether they have a normal distribution. For variables that do not conform to the normal distribution, the non-parametric Mann–Whitney Wilcoxon test is used. Secondly, information gain is used to determine the variables to be included in the Bayesian model. According to the results of information gain, R and Netica were used to build the Bayesian network model and implement Bayesian inference, respectively. Stata15.0 was used for data analysis and *p* < 0.05 was statistically significant.

## Results

### Demographics

Table 1 shows the demographics of the study participants. There were no significant differences between the PD and control group in sex, age, drinking, smoking, profession, and education level characteristics (*p* > 0.05).

**Table 1:** Basic characteristics of the study participants (*N* = 36).

Characteristics	PD (*n* = 18)	Control (*n* = 18)	*df*	*χ^2^*	*P*
Sex, *n* (%)			1	3.01	0.08
Male	9 (50.00)	4 (22.22)			
Female	9 (50.00)	14 (77.78)			
Age, *n* (%)			3	7.57	0.06
< 60	1 (5.56)	6 (33.33)			
60–69	5 (27.78)	7 (38.89)			
70–80	10 (55.56)	5 (27.78)			
> 80	2 (11.11)	0 (0.00)			
Drinking, *n* (%)	7 (38.89)	3 (16.67)	1	2.22	0.14
Smoking, *n* (%)	4 (22.22)	2 (11.11)	1	0.8	0.37
Profession, *n* (%)			2	1.13	0.57
Retired	6 (33.33)	4 (22.22)			
Farmer	10 (55.56)	13 (72.22)			
Worker	2 (1.11)	1 (5.56)			
Education level, *n* (%)			3	0.25	0.97
Primary school	10 (55.56)	11 (61.11)			
Middle school	3 (16.67)	3 (16.67)			
High school	3 (16.67)	2 (11.11)			
University	2 (11.11)	2 (11.11)			
H&Y, *n* (%)			2	10.29	0.001
Early stage (1–2.5)	10(55.56)	0 (0.00)			
Middle stage (3)	8(44.44)	0 (0.00)			
Advanced stage (4–5)	0(0.00)	0 (0.00)			
UPDRS, *n* (%)					
<30	8(44.44)	0 (0.00)	2	13.85	0.001
30–60	9(50.00)	0 (0.00)			
>60	1(5.56)	0 (0.00)			
Date, *n* (%)			2	10.29	0.006
<5	10(55.56)	0 (0.00)			
5–10	4(22.22)	0 (0.00)			
>10	4(22.22)	0 (0.00)			

PD, Parkinson’s disease; H&Y, Hoehn-Yahr scale; UPDRS, Unified Parkinson’s Disease Rating Scale; Date, Duration of disease, years; df, degrees of freedom.

### Phonation tests for three kinds of syllables

We analyzed the facial expressions in PD and control groups in terms of the type of syllable. The face parameters showed significant differences among three types of syllables (*p* < 0.001). The median values for happiness, surprise, valence, and arousal were significantly lower in the PD group, while the median values for negative expressions (unhappiness, anger, fear, and disgust) were significantly higher in the PD group for all three syllabic tests, a finding consistent with our previous study (11). Interestingly, the PD group presented lower median values for the expression of neutral feelings in the monosyllabic and disyllabic tests, whereas the median value of neutral feelings was higher in the PD group than the control group in the multisyllabic test (Table 2).

**Table 2:** Comparison of the face parameters for the Parkinson’s disease (PD) and control groups in the three syllabic tests.

Face parameter	Group	Monosyllabic test				Disyllabic test				Multisyllabic test			
		n	M (IQR)	Z	P	n	M (IQR)	Z	P	n	M (IQR)	Z	P
Neutral	PD	12,274	0.57 (0.24)	22.45	<0.001	7,927	0.59 (0.24)	−9.44	<0.001	8,189	0.62 (0.27)	−4.33	<0.001
	Control	16,258	0.64 (0.32)			12,104	0.62 (0.30)			10,472	0.60 (0.28)		
Happy	PD	12,274	0.03 (0.04)	58.80	<0.001	7,927	0.04 (0.05)	56.14	<0.001	8,189	0.05 (0.07)	43.09	<0.001
	Control	16,258	0.07 (0.15)			12,104	0.09 (0.17)			10,472	0.09 (0.19)		
Sad	PD	12,274	0.25 (0.18)	78.20	<0.001	7,927	0.26 (0.20)	62.96	<0.001	8,189	0.24 (0.22)	55.04	<0.001
	Control	16,258	0.11 (0.14)			12,104	0.11 (0.14)			10,472	0.10 (0.14)		
Angry	PD	12,274	0.03 (0.05)	61.91	<0.001	7,927	0.03 (0.05)	56.04	<0.001	8,189	0.053 (0.06)	44.67	<0.001
	Control	16,258	0.02 (0.02)			12,104	0.02 (0.02)			10,472	0.02 (0.02)		
Surprised	PD	12,274	0.02 (0.02)	57.62	<0.001	7,927	0.02 (0.02)	57.54	<0.001	8,189	0.02 (0.02)	59.16	<0.001
	Control	16,258	0.04 (0.04)			12,104	0.04 (0.05)			10,472	0.04 (0.06)		
Scared	PD	12,274	0.04 (0.05)	85.55	<0.001	7,927	0.04 (0.04)	75.60	<0.001	8,189	0.04 (0.05)	55.94	<0.001
	Control	16,258	0.02 (0.02)			12,104	0.02 (0.02)			10,472	0.02 (0.02)		
Disgusted	PD	12,274	0.05 (0.04)	42.11	<0.001	7,927	0.05 (0.04)	35.19	<0.001	8,189	0.04 (0.03)	24.11	<0.001
	Control	16,258	0.03 (0.03)			12,104	0.03 (0.04)			10,472	0.03 (0.04)		
Valence	PD	12,274	0.22 (0.20)	79.71	<0.001	7,927	0.24 (0.25)	67.67	<0.001	8,189	−0.21 (0.27)	55.95	<0.001
	Control	16,258	0.04 (0.24)			12,104	0.03 (0.26)			10,472	−0.02 (0.25)		
Arousal	PD	12,274	0.08 (0.18)	20.47	<0.001	7,927	0.06 (0.10)	41.88	<0.001	8,189	0.09 (0.16)	24.88	<0.001
	Control	16,258	0.13 (0.21)			12,104	0.14 (0.21)			10,472	0.15 (0.21)		

IQR, interquartile range; M, median.

### Transformed data

Because a Bayesian network can only be implemented with qualitative data, we divided the data for the PD and control groups based on 95% confidence intervals. For more details, please see the Supplementary Tables 1–4.

### Structure and parameters of the Bayesian network models

We applied information gain to implement feature selection with the R software. Supplementary Figure 1 shows the four Bayesian network models of the first 15 variables filtered by information gain to predict the probability of PD. In each model, the three variables that best distinguish PD are age, education level, and profession. The probability of PD increases with age (24) and a study in European populations found that men with more years of education are more likely to develop PD compared with women (25). Darweesh et al. (26) found that career choices are influenced by dopaminergic degeneration and the risk of PD varies dramatically based on occupation choice in midlife. In addition, among the face parameters, we found that “happy” was the most affective factor in the unsegmented-syllabic, monosyllabic, and disyllabic models, while arousal is the best predictor in the multisyllabic model.

### Model presentation in R and the degree of fit

Using the information gain results, we trained Bayesian Network models with the four different syllabic tests by using the bnlearn package of R software: (a) a tree-augmented Bayesian network with the entire dataset, (b) a tree-augmented Bayesian network with the monosyllabic test, (c) a tree-augmented Bayesian network with the disyllabic test, and (d) a tree-augmented Bayesian network with the multisyllabic test (Supplementary Figure 2). Each Bayesian model includes a parent node ‘Group’ of all child nodes and 15 child nodes, which include six demographic factors and nine facial expression variables. Each of these child nodes is limited to two parent nodes. Supplementary Figure 2 shows that the variable with the largest number of child nodes of the four models is the demographic factor age. There are four child nodes in the unsegmented-syllabic and multisyllabic models, five in the dissyllabic model, and seven in the monosyllabic model. This outcome suggests that age can directly influence the probability of PD and indirectly affect disease outcomes by affecting other aspects of the body. Education level and profession have a similar effect, but there are not as many child nodes as age. Valence, sad, and neutral also have child nodes through which they indirectly affect ‘Group’.

According to the information gain results, we trained a tree-augmented Bayesian network classifier. Table 3 shows the sensitivity, specificity, and area under the curve (AUC) of the training and test sets for the four models. The AUC for the training and tests sets are all close to 0.96, with no significant differences between the models. Based on these findings, the multisyllabic and monosyllabic models have the greatest sensitivity.

**Table 3:** The specificity, sensitivity, and area under the curve of each data set.

Model	Sensitivity (%)		Specificity (%)		AUC	
	Training	Test	Training	Test	Training	Test
Monosyllabic	0.98	0.98	0.94	0.95	0.960	0.963
Disyllabic	0.95	0.96	0.96	0.96	0.958	0.961
Multisyllabic	0.98	0.98	0.94	0.94	0.962	0.960
Unsegmented syllabic	0.96	0.96	0.95	0.95	0.953	0.951

AUC, Area under the curve.

#### Model presentation in Netica

**Figure 2:**
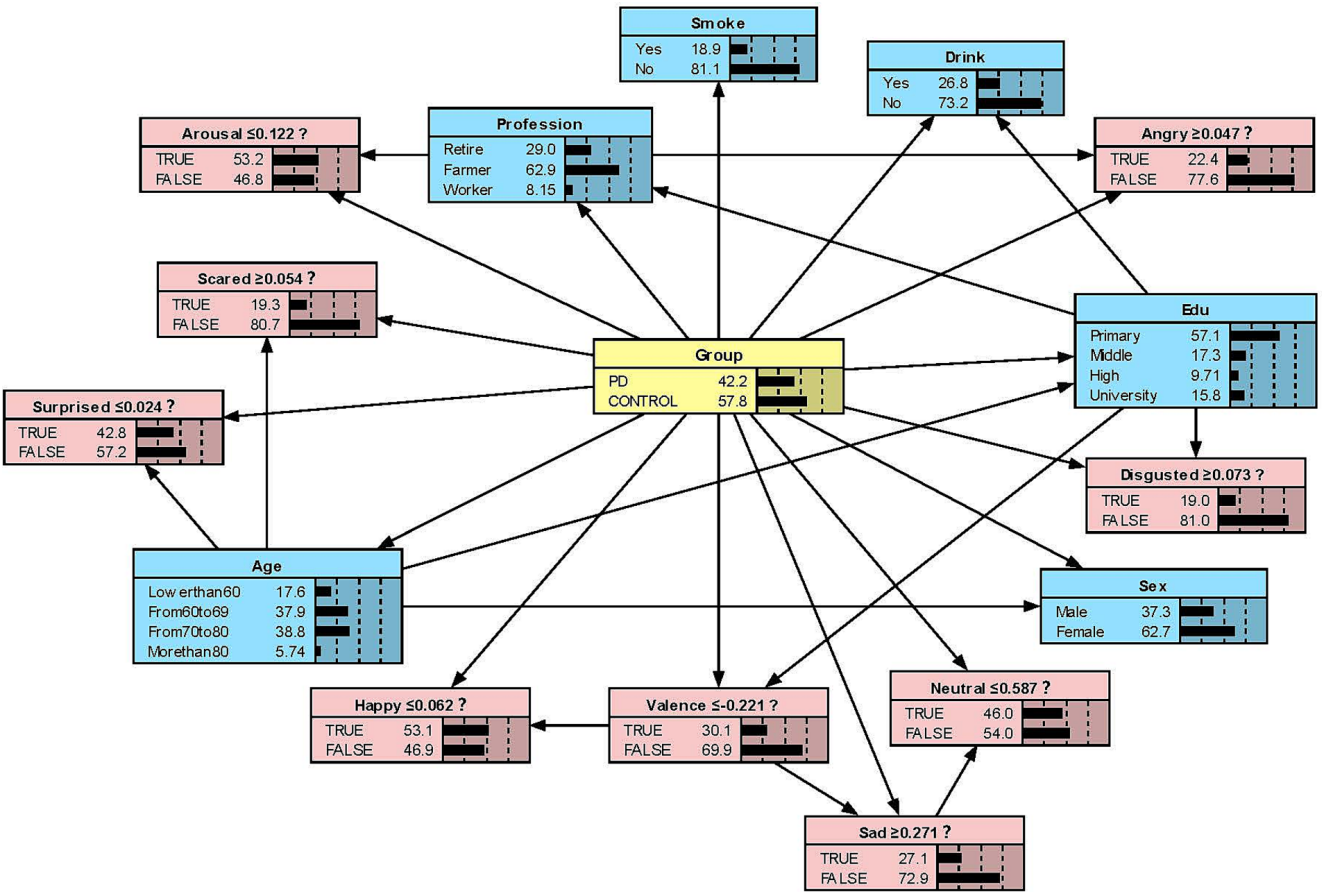
The Bayesian network model to predict the probability of Parkinson’s disease based on the unsegmented-syllabic model.

**Figure 3:**
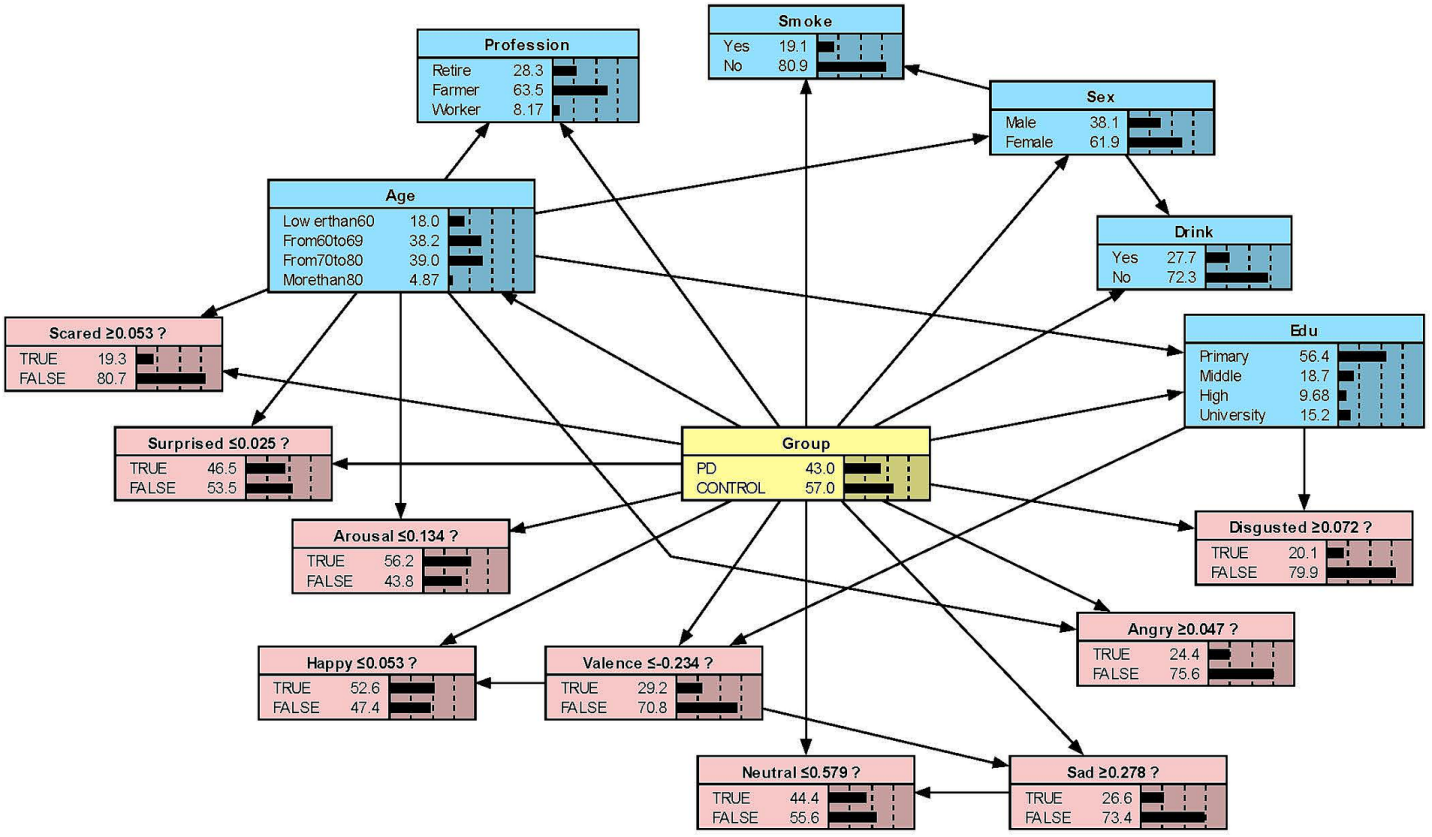
The Bayesian network model to predict the probability of Parkinson’s disease based on the monosyllabic model.

**Figure 4:**
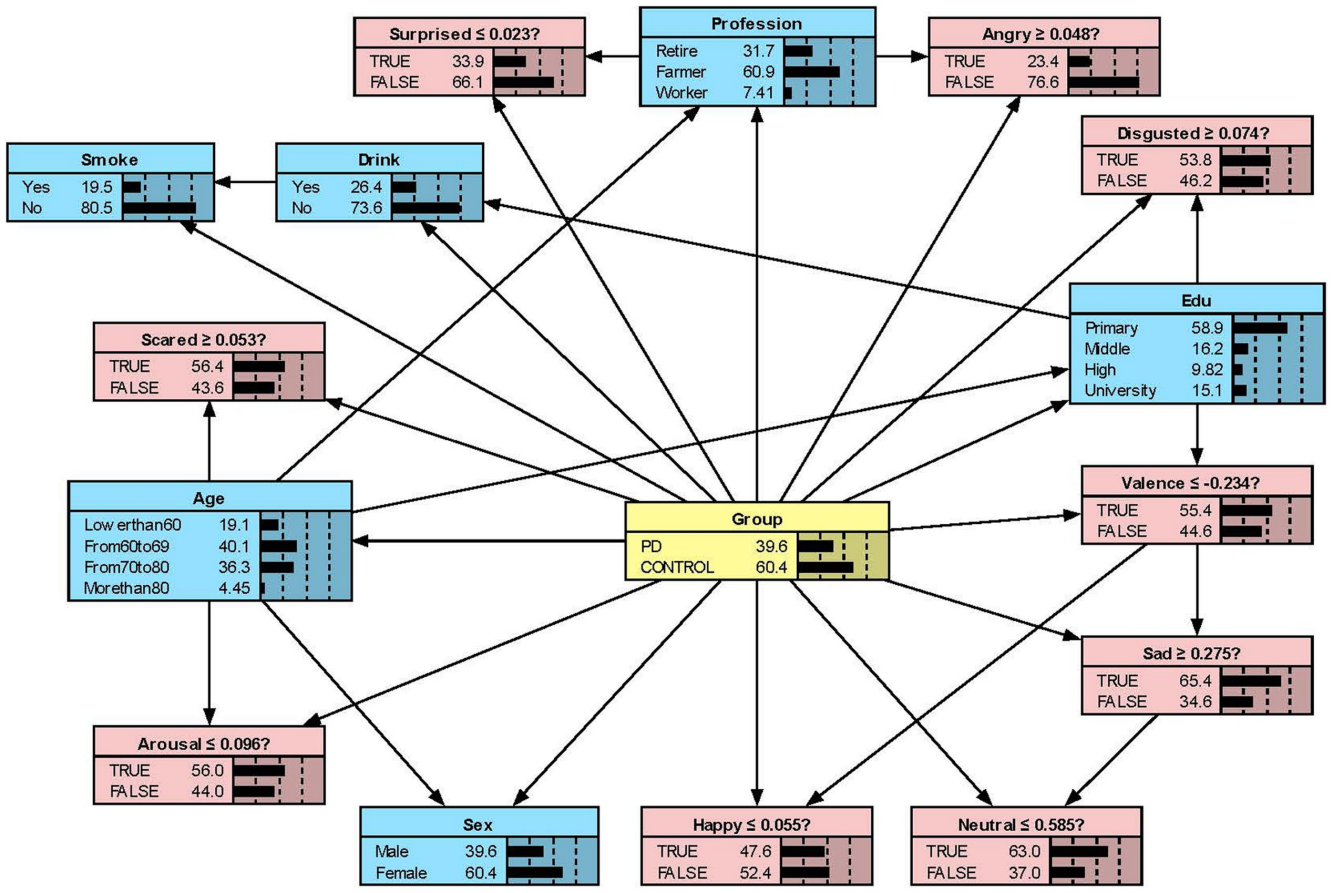
The Bayesian network model to predict the probability of Parkinson’s disease based on the disyllabic model.

**Figure 5:**
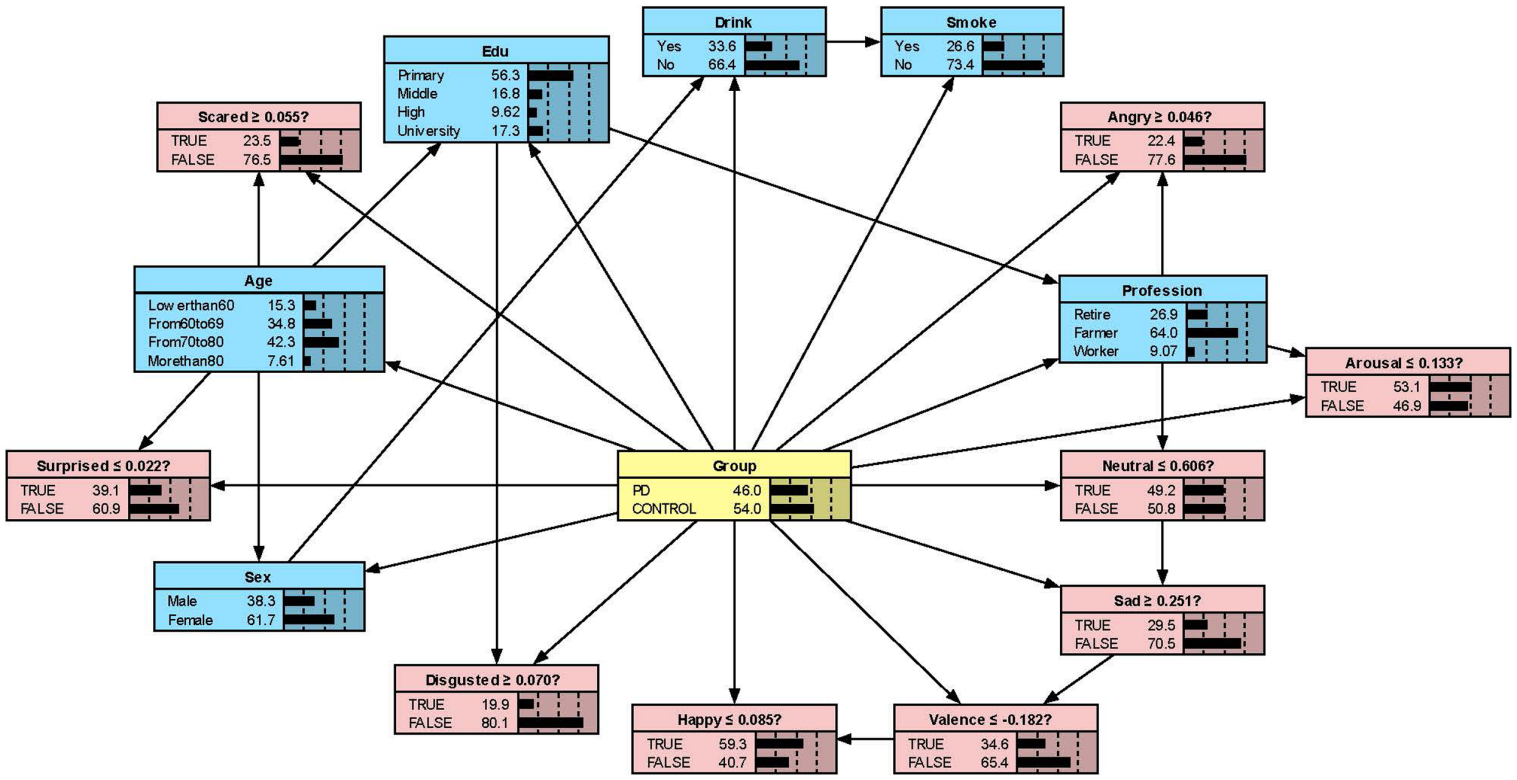
The Bayesian network model to predict the probability of Parkinson’s disease based on the multisyllabic model.

We constructed the Bayesian network models with Netica to obtain Figures 2–5. We derived the model layout from Supplementary Figure 2 (the results from the R bnlearn package). We found that in all syllabic tests, anger has a great impact on the posterior probability of PD. Meanwhile, in the unsegmented-syllabic, monosyllabic, and multisyllabic models, sad, scared facial expressions and valence greatly influence the posterior probability of PD, while neutral, happy, surprised, disgusted, and arousal facial expression have significantly less influence on the posterior probability of PD. Moreover, in the monosyllabic and unsegmented-syllabic models, the scared facial expression has the greatest influence, while the angry facial expression has the greatest influence for the disyllabic model. (Table 4).

**Table 4:** The prior and posterior probability of Parkinson’s disease.

Variable	Monosyllabic (%)	Disyllabic (%)	Multisyllabic (%)	Unsegmented syllabic (%)
Prior	43.00	39.60	46.00	42.20
Neutral	49.40	31.00	44.90	45.40
Happy	55.30	48.60	55.60	55.00
Sad	67.50	28.50	72.90	69.40
Angry	74.00	75.10	79.80	78.50
Surprised	56.00	67.10	63.50	57.70
Scared	84.70	25.40	80.30	82.40
Disgusted	49.70	13.90	48.00	46.50
Valence	69.60	36.50	71.60	70.70
Arousal	48.40	46.80	53.70	50.60

## Discussion

Numerous studies have demonstrated that Bayesian network models are more efficient and reliable compared with traditional frequentist approaches (27). By visually linking variables based on medical data, a Bayesian network can provide explicable results to determine a medical condition (28). Zhu et al. (29) showed that when different vowels were pronounced, the energy map of the high-density surface electromyography (HD sEMG) showed that the high-intensity areas appeared on different muscles. In addition, the clinical features of PD include facial bradykinesia and facial masking, which affects the contraction ability of the patient’s facial muscles, involuntary muscle movement and its susceptibility or stiffness (30). So, different facial muscles participate in the formation of different expressions when different vowels are pronounced, and the Noldus FaceReader can accurately record this process. In this study, we established Bayesian network models to predict the probability of PD based on facial expressions and demographic characteristics. These models provide clinicians with a validated decision-making tool to support the choice of diagnostic strategies in PD.

A Bayesian network model can clearly show the ‘parent’ and ‘child’ variables and their direct or indirect relationships (31). Regarding the direct or indirect relationships, we observed that ‘Group-Edu’, ‘Group-Disgusted’, and ‘Group-Edu-Disgusted’ connections in all four models, indicating that Edu and the disgusted facial expression are directly related to the probability of PD. Moreover, the disgusted facial expression is indirectly connected to the outcome. Our findings are like those reported by Ille et al. (32) people with PD have more problems controlling and expressing feelings of disgust than controls.

While previous studies have only objectively described facial expressions in people with PD, we aimed to determine the extent to which different facial expressions affect the prediction of disease. Our research found that all positive expressions were negatively associated with the probability of PD in the posterior probability of all syllables, except for valence in the disyllabic model. Negative expressions including sadness, anger, fear, and disgust in the unsegmented-syllabic, monosyllabic, multisyllabic models were positively related to the probability of PD, as well as anger in disyllabic model. For example, in the monosyllabic model, if we select the ‘false’ state of the variable happy—sat this point the happy score is higher than the boundary value – then we can observe the posterior probability of PD decreases. If the ‘true’ state of the variable scared is determined—the scared score is higher than the boundary value – then the posterior probability of PD increases.

Hypomimia can be misdiagnosed as depression, a nonmotor symptom of PD (33).However, studies have shown that masked face is one of the main early symptoms of Parkinson’s disease (34), and our previous research has shown that positive expressions are reduced and negative expressions are increased in PD patients, which was consistent with the results of our study on the facial performance of patients during monosyllable and multisyllabic pronunciation (11, 34). The articulatory feedback hypothesis (AFH) can reasonably explain this, based on the assumption that oral facial muscle tissue is also activated during articulation (35). Different vowels activate different facial muscles, and different facial muscles activate different expressions. For example, activation of the orbicularis oris muscle has a negative effect on facial expression, and activation of the zygomatic major muscle has a positive effect on facial expression (36).

As the complexity of test syllables increases, the cognitive load of PD patients also increases, making it difficult for the brain nerves to control muscle movement (37). PD patients may require more complex motion planning to produce accurate speech output. This includes more precise coordinated movements of the lip, tongue, and throat muscles (38). When we have a scared expression, most people’s mouths open in an “O” shape, which is the same as when monosyllabic “a,” “o,” and “e” (vowels) are pronounced. This is also one of the reasons why scared expressions increase in monosyllabic tests. When in a state of fear, individuals will contract specific facial muscles, such as the medial frontalis and frown muscles, thereby enlarging the eyes and nose (39). Anterior insula may also have a neural correlation with fearful facial expressions (40). Parkinson’s disease patients may experience structural and functional changes in brain regions such as the insula, amygdala, and ventral striatum, which can affect the expression of scared emotions. Studies have shown that PD patients activate additional neural network regions in their brains to support speech production and comprehension when performing complex speech tasks (41, 42). These additional neural network regions may represent compensation mechanisms for patients. Understanding the changes in cognitive load, motor planning requirements, and compensatory mechanisms of PD patients under different levels of speech complexity can help develop more effective rehabilitation treatment plans.

Much of the current research focuses on voluntary (posed) facial expressions (43). However, in terms of overall movement, speed, and timing, it is still unknown whether a posed expression is equivalent to an involuntary (spontaneous) expression. Therefore, using a voice test as a trigger to induce changes in facial expressions is more closely related to the autonomous state. Besides, Amratajska et al. (43) found that patients with PD with left-side onset were much slower than those with right-side onset in initiating angry and happy facial expressions, which means different areas of impairment of the brain may influence facial expressions in people with PD. Moreover, because only levodopa was stopped before the study, the effects of dopamine replacement therapy on masked face may still exist (8). This eventuality can be investigated in future studies.

Current research shows that facial expression analysis can be used as a non-invasive tool for detecting PD, only requiring a webcam or a phone with a camera (44). Thus, the bayes model incorporating facial expression and demographic variables may have clinical significance. In contrast to a thorough clinical assessment, a short and easy-to-administer screening tool may be useful. In future, the innovative approach that one can take through mobile technology (e.g., home training of rhythm skills using a dedicated app on a tablet device) would be a valuable complement to traditional therapeutic approaches (45).

Due to the limited by sample size in single research centre, this may contribute the selection bias. Furthermore, all candidates paused taking levodopa on the morning of the sound test, they continued to take other anti-Parkinson’s drugs. The sound test was not measured when the patient had severe motor symptoms according to the safety considerations. However, the used of other drugs may have affected the results, which we will further verify in future studies.

## Conclusion

We identified the facial expressions with a significant influence on the probability of PD. Except for sad, scared, and disgusted generated by reading disyllables, negative expressions generated by reading other syllables were positively associated with the probability of PD. In addition, scared expressions produced during monosyllabic reading had the greatest effect on the probability of PD, and disgusted expressions produced during multisyllabic reading had the least effect. Our research employed Bayesian network model as the potential predictive tool for Parkinson’s disease for the purpose of providing a preliminary foundation for clinical diagnosis and treatment.

## Data Availability

The original contributions presented in the study are included in the article/Supplementary material, further inquiries can be directed to the corresponding authors.

## Supplementary material

SUPPLEMENTARY FIGURE 1 Top 15 variables to predict the prevalence of Parkinson’s disease. The Bayesian network models are shown for **(a)** the unsegmented-syllabic test, **(b)** the monosyllabic test, **(c)** the disyllabic test, and **(d)** the multisyllabic test.

SUPPLEMENTARY FIGURE 2 The Bayesian network models from R of **(a)** the unsegmented-syllabic test**, (b)** the monosyllabic test, **(c)** the disyllabic test, and **(d)** the multisyllabic test.

SUPPLEMENTARY FIGURE 3 Known network changes based on the happy facial expression.

SUPPLEMENTARY FIGURE 4 Known network changes based on the scared facial expression.
