# Supplementary figures and images for: Changes in facial expressions can distinguish Parkinson’s disease via Bayesian inference
Source: Front Neurol. 2025 Mar 27;16:1533942. doi: 10.3389/fneur.2025.1533942 (PMC11983656; doi:10.3389/fneur.2025.1533942)

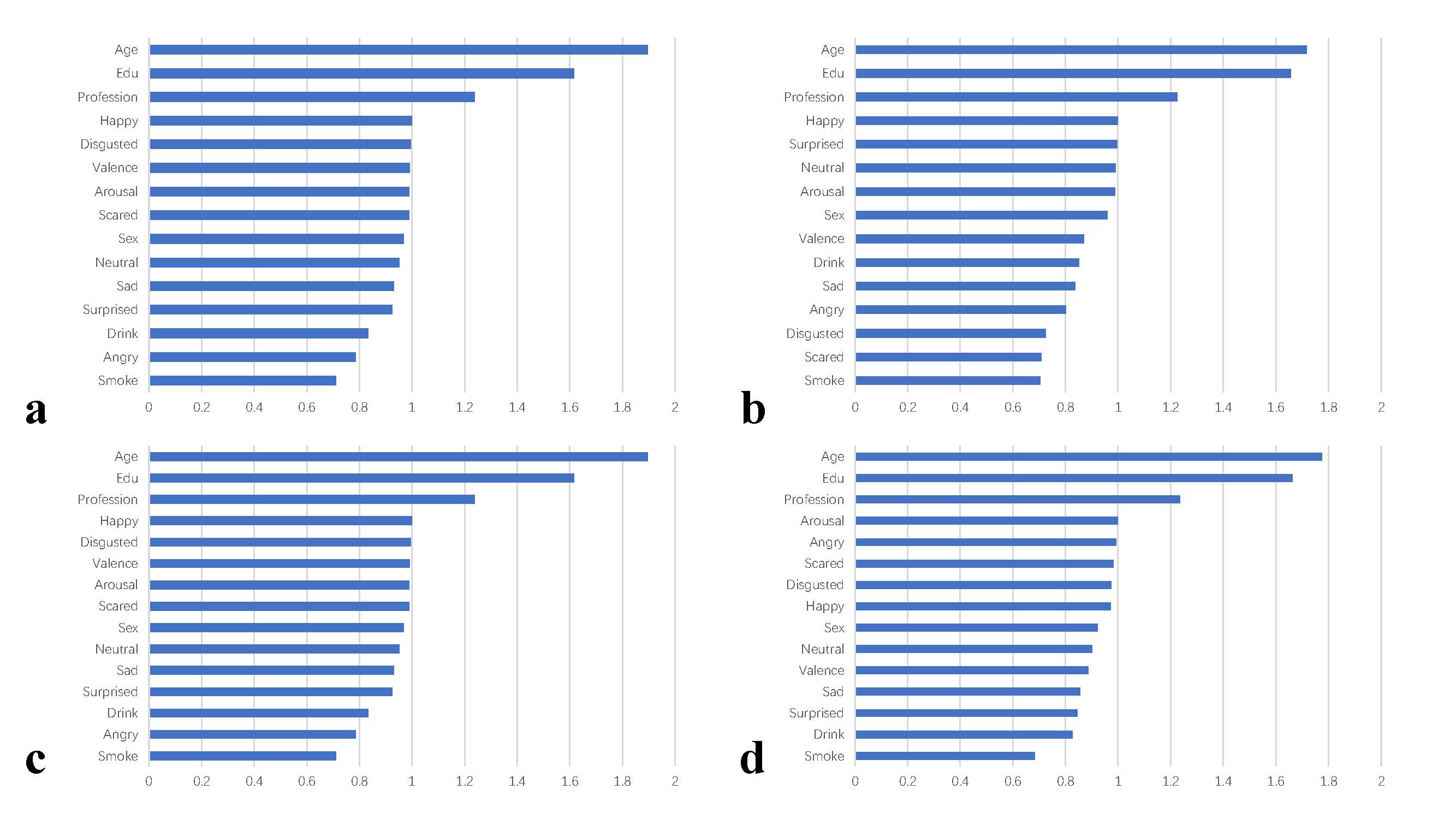

Supplement: SUPPLEMENTARY FIGURE 1 — Top 15 variables to predict the prevalence of Parkinson’s disease. The Bayesian network models are shown for (a) the unsegmented-syllabic test, (b) the monosyllabic test, (c) the disyllabic test, and (d) the multisyllabic test. [file Image_1.JPEG]

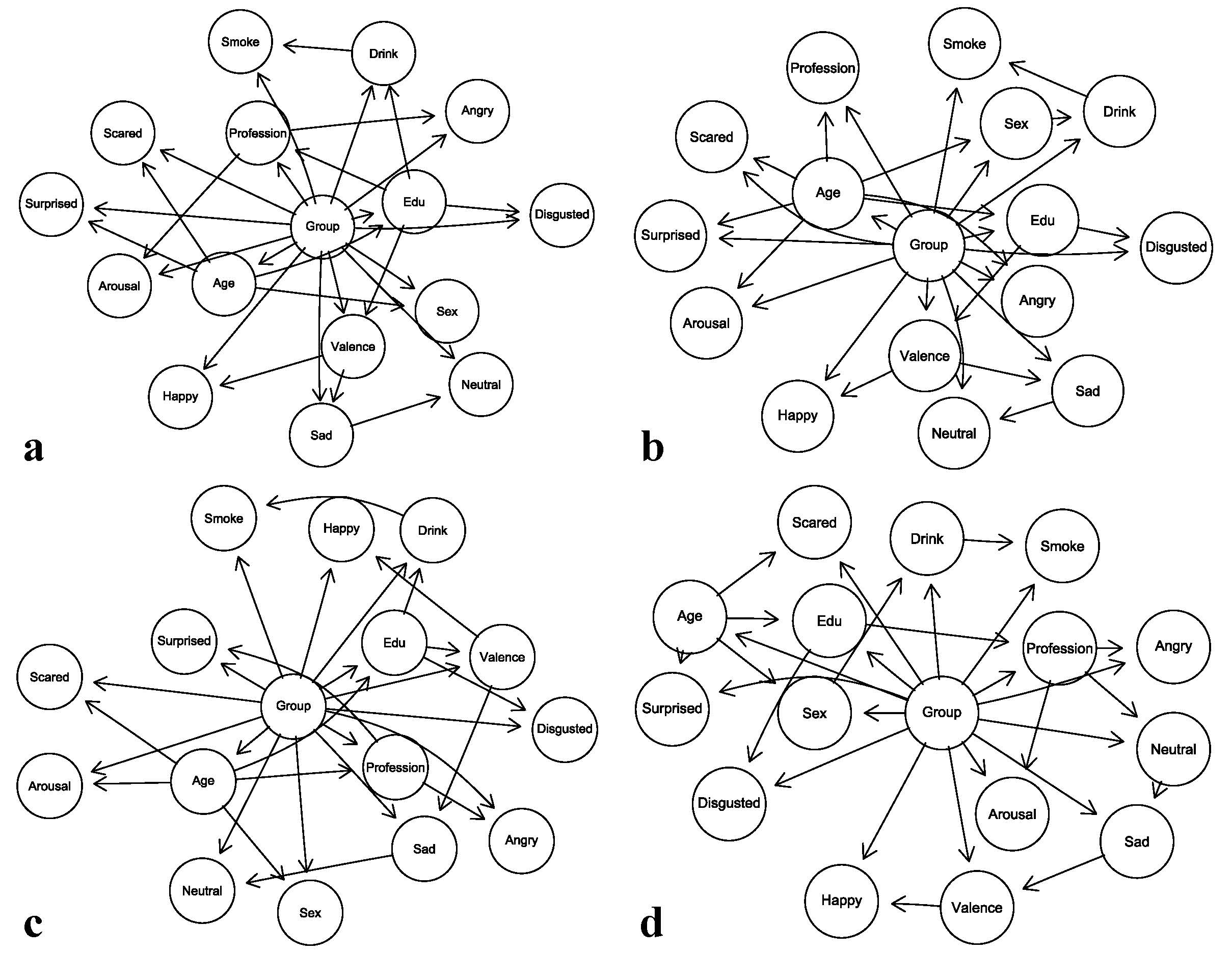

Supplement: SUPPLEMENTARY FIGURE 2 — The Bayesian network models from R of (a) the unsegmented-syllabic test, (b) the monosyllabic test, (c) the disyllabic test, and (d) the multisyllabic test. [file Image_2.JPEG]

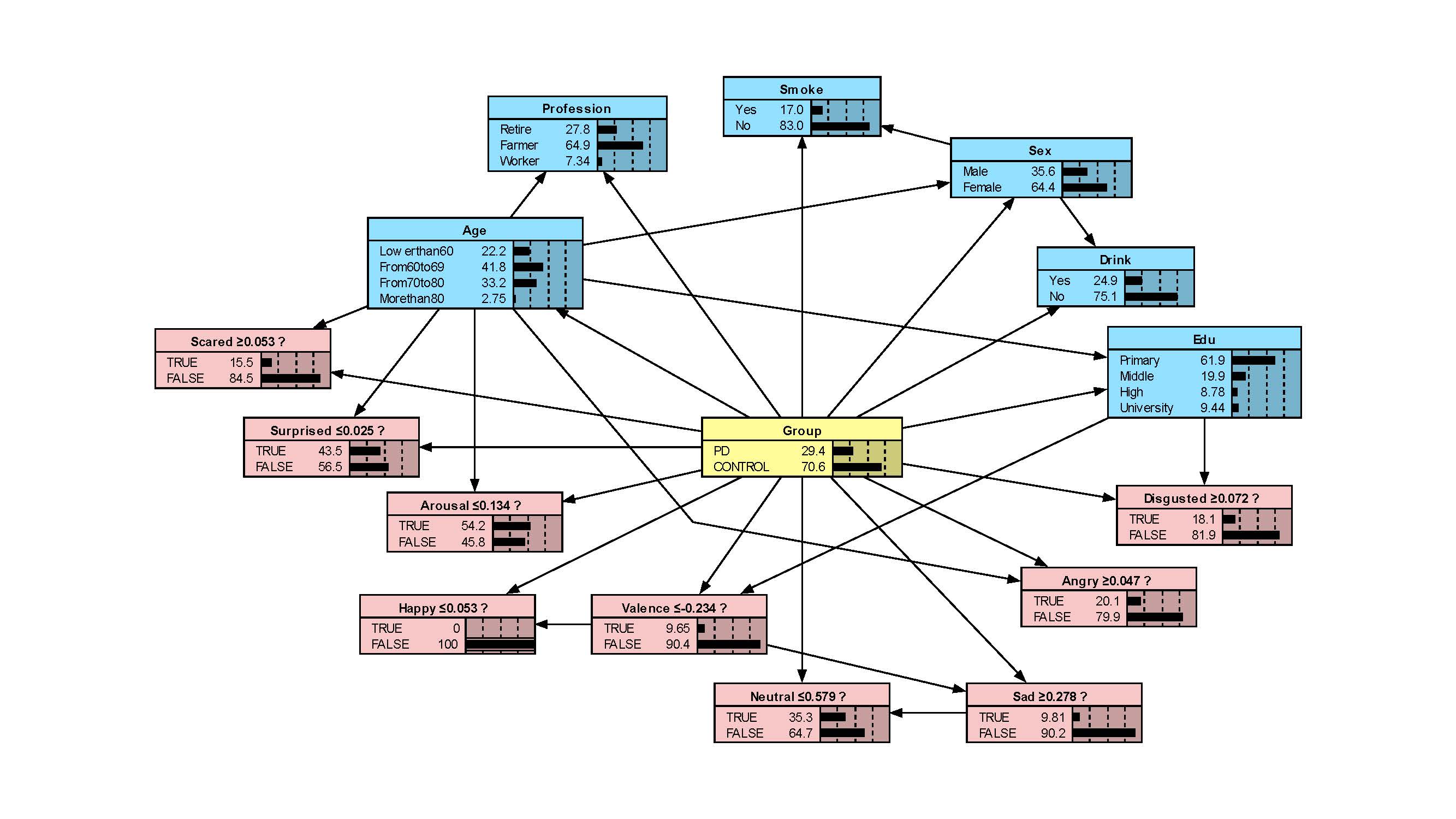

Supplement: SUPPLEMENTARY FIGURE 3 — Known network changes based on the happy facial expression. [file Image_3.JPEG]

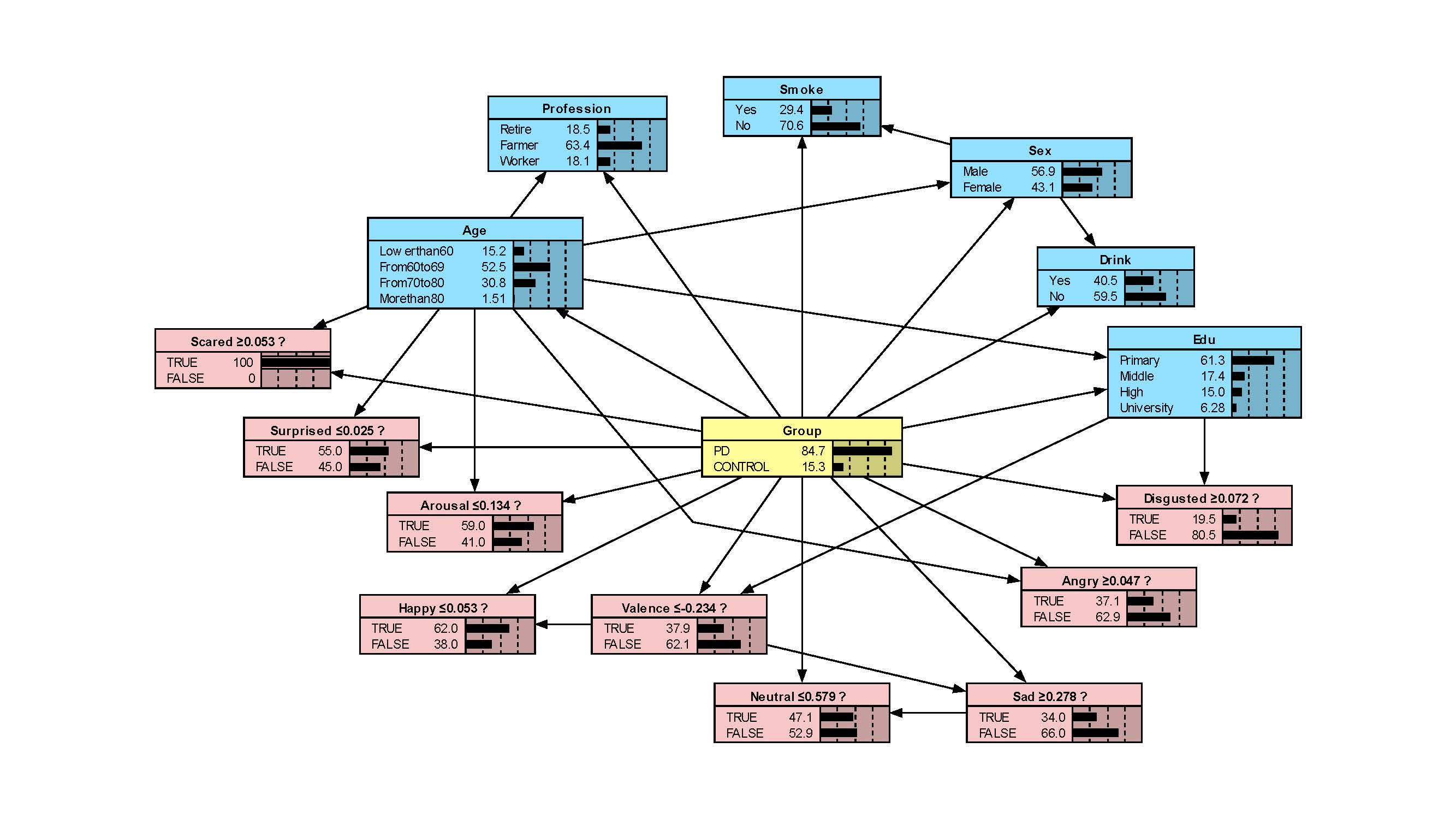

Supplement: SUPPLEMENTARY FIGURE 4 — Known network changes based on the scared facial expression. [file Image_4.JPEG]
