# Supplementary material for: Changes in facial expressions can distinguish Parkinson’s disease via Bayesian inference
Source: Front Neurol. 2025 Mar 27;16:1533942. doi: 10.3389/fneur.2025.1533942 (PMC11983656; doi:10.3389/fneur.2025.1533942)
Supplement: Supplementary file 5 [file Table_1.DOCX]

**Supplementary Table 1** Assignment of latent factors for clinical data in the monosyllabic model.

| **Variable** | **Definition** | **Assignment** |
| --- | --- | --- |
| **Group (PD or not)** | Y | Yes = 1, No = 0 |
| **Age** | X1 | < 60 = 1, 60~69 = 2, 70~80 = 3, > 80 = 4 |
| **Gender** | X2 | Male = 1, Female = 2 |
| **Profession** | X3 | Retire = 1, Farmer = 2, Worker = 4 |
| **Drink** | X4 | Yes = 1, No = 0 |
| **Smoke** | X5 | Yes = 1, No = 0 |
| **Edu** | X6 | Primary = 1, Middle = 2, High = 3, University = 4 |
| **Neutral** | X7 | ≤ 0.579 = 1, > 0.579 = 0 |
| **Happy** | X8 | ≤ 0.053 = 1, > 0.053 = 0 |
| **Sad** | X9 | ≥ 0.278 = 1, < 0.278 = 0 |
| **Angry** | X10 | ≥ 0.047 = 1, < 0.047 = 0 |
| **Surprised** | X11 | ≤ 0.025 = 1, > 0.025 = 0 |
| **Scared** | X12 | ≥ 0.053 = 1, < 0.053 = 0 |
| **Disgusted** | X13 | ≥ 0.072 = 1, < 0.072 = 0 |
| **Valence** | X14 | ≤ -0.234 = 1, > -0.234 = 0 |
| **Arousal** | X15 | ≤ 0.134 = 1, > 0.134 = 0 |

**Abbreviations:** Edu, education level; PD, Parkinson’s disease.

**Supplementary Table 2** Assignment of latent factors for clinical data in the disyllabic model.

| **Variable** | **Definition** | **Assignment** |
| --- | --- | --- |
| **Group (PD or not)** | Y | Yes = 1, No = 0 |
| **Age** | X1 | < 60 = 1, 60~69 = 2, 70~80 = 3, > 80 = 4 |
| **Gender** | X2 | Male = 1, Female = 2 |
| **Profession** | X3 | Retire = 1, Farmer = 2, Worker = 4 |
| **Drink** | X4 | Yes = 1, No = 0 |
| **Smoke** | X5 | Yes = 1, No = 0 |
| **Edu** | X6 | Primary = 1, Middle = 2, High = 3, University = 4 |
| **Neutral** | X7 | ≤ 0.585 = 1, > 0.585 = 0 |
| **Happy** | X8 | ≤ 0.055 = 1, > 0.055 = 0 |
| **Sad** | X9 | ≥ 0.275 = 1, < 0.275 = 0 |
| **Angry** | X10 | ≥ 0.048 = 1, < 0.048 = 0 |
| **Surprised** | X11 | ≤ 0.023 = 1, > 0.023 = 0 |
| **Scared** | X12 | ≥ 0.053 = 1, < 0.053 = 0 |
| **Disgusted** | X13 | ≥ 0.074 = 1, < 0.074 = 0 |
| **Valence** | X14 | ≤ -0.234 = 1, > -0.234 = 0 |
| **Arousal** | X15 | ≤ 0.096 = 1, > 0.096 = 0 |

**Abbreviations:** Edu, education level; PD, Parkinson’s disease.

**Supplementary Table 3**. Assignment of latent factors for clinical data in the multisyllabic model.

| **Variable** | **Definition** | **Assignment** |
| --- | --- | --- |
| **Group (PD or not)** | Y | Yes = 1, No = 0 |
| **Age** | X1 | < 60 = 1, 60~69 = 2, 70~80 = 3, > 80 = 4 |
| **Gender** | X2 | Male = 1, Female = 2 |
| **Profession** | X3 | Retire = 1, Farmer = 2, Worker = 4 |
| **Drink** | X4 | Yes = 1, No = 0 |
| **Smoke** | X5 | Yes = 1, No = 0 |
| **Edu** | X6 | Primary = 1, Middle = 2, High = 3, University = 4 |
| **Neutral** | X7 | ≤ 0.606 = 1, > 0.606 = 0 |
| **Happy** | X8 | ≤ 0.085 = 1, > 0.085 = 0 |
| **Sad** | X9 | ≥ 0.251 = 1, < 0.251 = 0 |
| **Angry** | X10 | ≥ 0.046 = 1, < 0.046 = 0 |
| **Surprised** | X11 | ≤ 0.022 = 1, > 0.022 = 0 |
| **Scared** | X12 | ≥ 0.055 = 1, < 0.055 = 0 |
| **Disgusted** | X13 | ≥ 0.070 = 1, < 0.070 = 0 |
| **Valence** | X14 | ≤ -0.182 = 1, > -0.182 = 0 |
| **Arousal** | X15 | ≤ 0.133 = 1, > 0.133 = 0 |

**Abbreviations:** Edu, education level; PD, Parkinson’s disease.

**Supplementary Table 4** Assignment of latent factors for clinical data in the unsegmented-syllable model.

| **Variable** | **Definition** | **Assignment** |
| --- | --- | --- |
| **Group (PD or not)** | Y | Yes = 1, No = 0 |
| **Age** | X1 | < 60 = 1, 60~69 = 2, 70~80 = 3, > 80 = 4 |
| **Gender** | X2 | Male = 1, Female = 2 |
| **Profession** | X3 | Retire = 1, Farmer = 2, Worker = 4 |
| **Drink** | X4 | Yes = 1, No = 0 |
| **Smoke** | X5 | Yes = 1, No = 0 |
| **Edu** | X6 | Primary = 1, Middle = 2, High = 3, University = 4 |
| **Neutral** | X7 | ≤ 0.587 = 1, > 0.587 = 0 |
| **Happy** | X8 | ≤ 0.062 = 1, > 0.062 = 0 |
| **Sad** | X9 | ≥ 0.271 = 1, < 0.271 = 0 |
| **Angry** | X10 | ≥ 0.047 = 1, < 0.047 = 0 |
| **Surprised** | X11 | ≤ 0.024 = 1, > 0.024 = 0 |
| **Scared** | X12 | ≥ 0.054 = 1, < 0.054 = 0 |
| **Disgusted** | X13 | ≥ 0.073 = 1, < 0.073 = 0 |
| **Valence** | X14 | ≤ -0.221 = 1, > -0.221 = 0 |
| **Arousal** | X15 | ≤ 0.122 = 1, > 0.122 = 0 |

**Abbreviations:** Edu, education level; PD, Parkinson’s disease.
